# Progressive patterns of neurological disability in multiple sclerosis and neuromyelitis optica spectrum disorders

**DOI:** 10.1038/s41598-020-70919-w

**Published:** 2020-08-17

**Authors:** Tetsuya Akaishi, Toshiyuki Takahashi, Tatsuro Misu, Michiaki Abe, Tadashi Ishii, Juichi Fujimori, Masashi Aoki, Kazuo Fujihara, Ichiro Nakashima

**Affiliations:** 1 Department of Neurology, Tohoku University Graduate School of Medicine, Seiryo-machi 1-1, Aoba-ku, Sendai, Miyagi 980-8574 Japan; 2 Department of Education and Support for Regional Medicine, Tohoku University Hospital, Sendai, Japan; 3 Department of Neurology, National Hospital Organization Yonezawa National Hospital, Yonezawa, Japan; 4 Department of Neurology, Tohoku Medical and Pharmaceutical University, Sendai, Japan; 5 Department of Multiple Sclerosis Therapeutics, Fukushima Medical University, Fukushima, Japan

**Keywords:** Neurology, Multiple sclerosis

## Abstract

The progressive patterns of neurological disability in multiple sclerosis (MS) and neuromyelitis optica spectrum disorders (NMOSD) and the significance of clinical relapses to the progressions of neurological disability in these diseases have not been fully elucidated. In this study, to elucidate the impact of relapses to the progression of accumulated neurological disability and to identify the factors to affect the progression of neurological disability in MS and NMOSD, we followed 62 consecutive MS patients and 33 consecutive NMOSD patients for more than 5 years with the clinical symptoms, relapse occurrence, and Expanded Disability Status Scale (EDSS) in the chronic phase. All enrolled MS patients were confirmed to be negative for serum anti-myelin oligodendrocyte glycoprotein antibody. As a result, patients with NMOSD showed significantly severer neurological disability at 5 years from onset than MS patients. Progression in EDSS score was almost exclusively seen after clinical attacks in NMOSD, whereas progression could be observed apart from relapses in MS. Neurological disability did not change without attacks in NMOSD, whereas it sometimes spontaneously improved or deteriorated apart from relapses in MS (p < 0.001). In patients with MS, those with responsible lesions primarily in spinal cord were more likely to show such spontaneous improvement. In conclusion, clinical deterioration in NMOSD patients is irreversible and almost exclusively takes place at the timing of clinical attacks with stepwise accumulation of neurological disability. Meanwhile, changes in EDSS score can be seen apart from relapses in MS patients. Neurological disability in MS patients is partly reversible, and the patients with disease modifying drugs sometimes present spontaneous improvement of the neurological disability.

## Introduction

Multiple sclerosis (MS) and neuromyelitis optica spectrum disorders (NMOSD) are major autoimmune-related neurological diseases that predominantly impairs the central nervous system (CNS) but have distinct pathophysiological mechanisms^1,2^. Both diseases typically present recurrent clinical attacks with lesions in cerebrum, optic nerves, brainstem, and spinal cord^1,3^. At present, MS is diagnosed based on the dissemination of lesions in time and space^4–6^, whereas NMOSD is diagnosed both by the clinical history and the presence of serum anti-aquaporin-4 antibody (AQP4-IgG)^1,7^.

The accumulation of neurological impairment in MS is thought to be mainly comprised of the following two components: subclinical progressive brain atrophy and recurrent clinical relapses during which the responsible lesions are contrast-enhanced in MRI^8^. Based on this concept, clinical course of MS is generally categorized into the following three subtypes: primary progressive MS (PPMS), relapsing–remitting MS (RRMS), and secondary progressive MS (SPMS)^9,10^. Meanwhile, progressive pattern of neurological impairment in NMOSD has not been so much studied until now, although the subsequent neurological disability is generally severer in NMOSD than in MS^11–13^. From before, the progression of neurological disability in NMOSD has been empirically thought to occur mainly at the timing of clinical attacks^14^, but whether it occurs even during the intermittent period between attacks or not has not been proved yet. Because the expected clinical course will surely affect the achieved outcomes in clinical studies enrolling the patients with these diseases, elucidating the characteristic clinical course and factors that affect the progression of neurological disability in these diseases has many clinical significances.

In this study, we enrolled an enough number of MS and NMOSD patients who have their clinical onset between 2000 and 2015, and followed their neurological disability every year from the first visit to our hospital. The progressive pattern of their neurological disability was evaluated with other information such as patient background and relapses to identify the clinical factors that mainly regulate the progression of neurological disability in each of MS and NMOSD.

## Methods

### Patients

For this study, a total of 62 consecutive MS patients and 33 consecutive AQP4-IgG-positive NMOSD patients, who had their clinical onset between 2000 and 2015 and treated in our university hospital were initially collected. These initial cohorts were prospectively followed up, among which 57 MS patients (91.9%) and 31 NMOSD patients (93.9%) were followed and treated in our university hospital for more than 5 years from their onset. All enrolled MS patients were confirmed to be negative for the presence of serum anti-myelin oligodendrocyte glycoprotein (MOG) antibody by utilizing the cell-based assay method^15^. The presence of serum AQP4-IgG in the enrolled NMOSD patients was also confirmed based on the cell-based assay method^16,17^.

All of the 5 MS patients who was not followed for more than 5 years dropped out because of moving. One of the 2 NMOSD patients who was not followed for more than 5 years died because of a malignant tumor 2 years after the onset of NMOSD, but another patient dropped out because of moving.

### Collected data

In these patients, comprehensive clinical information was collected, such as onset age, sex, follow up period as of 2019, types of relapse preventive therapies, type and timing of clinical attacks, and the titer of serum anti-AQP4 antibody. The irreversible neurological impairment was evaluated with the Expanded Disability Status Scale (EDSS) in every 1 or 2 years in these patients^18^. Because neurological disability can drastically exacerbate and recover in the acute and subacute phases of clinical relapses with proper treatments with immune-suppressants, EDSS scores within 3 months from the last relapse were not used in this study. Relapses in MS and NMOSD were defined by the presence of clinically evidenced gadolinium-enhanced T1-weighted lesions with the neurological symptoms sustained for more than 24 h in the absence of fever or infection^4^.

As for other clinical information, data about the number of cerebral lesions at 5 years from the onset and the site of lesion responsible for the neurological disability (i.e. cerebral, optic nerves, brainstem, spinal cord) were also collected in MS patients. To identify the factors that affect the neurological disability in MS, we also evaluated the gray matter volume and white matter lesion volume in some of them with a volumetric method as previously reported^19^.

### Statistical analysis

Comparisons of two distributions were performed with either of the Student’s t-test or Mann–Whitney U test, based on the normality of distributions. Comparisons of frequencies were performed with either of the chi-squared test or Fisher’s exact test, based on the size in each cell. A value of p < 0.05 was regarded to be statistically significant. The analyses were conducted using either SPSS Statistics Base 22 software (IBM, Armonk, NY, USA) or MATLAB R2015a (MathWorks, Natick, MA, USA).

### Ethical approval

This study was approved by the institutional review board of the Tohoku University Graduate School of Medicine (approval number: 2010589), and was carried out in accordance with the standards stated in the 1964 Declaration of Helsinki and its later amendments or comparable ethical standards.

### Informed consent

Informed consent was obtained from all individual participants included in the study.

## Results

### Cohort demographics

The total follow-up period with EDSS evaluation in the initially collected MS patients with onset between 2000 and 2015 was 337 person-year (62 patients) as of Oct 2019; that in the initially collected NMOSD patients with onset between 2000 and 2015 was 229 person-year (33 patients). Patients’ background in both groups are listed in Table 1 and compared between the groups. As for therapeutic interventions, in MS group, 58 of the 62 patients have been treated with disease modifying drugs (DMDs), such as interferon (IFN) beta, glatiramer acetate, fingolimod, or natalizumab. In the 58 patients with DMDs, 26 were initially treated with IFN beta, but later changed DMD to fingolimod. Other 2 patients have been treated with oral prednisolone (PSL) and the remaining 2 patients are untreated. In NMOSD group, 32 of the 33 patients were treated with low-dose oral PSL (< 20 mg/day) with or without other immune-suppressants (i.e. azathioprine, cyclosporine, tacrolimus). The remaining 1 NMOSD patient has not been treated with any kind of relapse preventive therapies.

**Table 1:** Patient background and clinical course in MS and NMOSD.

	MS (n = 62)	NMOSD (n = 33)	p-value
Male:Female	12:50	3:30	0.25
Onset age (mean ± SD)	29.9 ± 8.6	48.5 ± 13.8	< 0.0001
Total EDSS follow-up period (person-year)	337	229	–
EDSS follow-up years per capita (median, IQR)	6 (3–7)	7 (5–9)	0.0460
**Cross-sectional EDSS**			
EDSS at 5 years from onset (median, IQR)	1.5 (1.0–2.0; n = 53)	3.0 (2.0–5.0; n = 31)	< 0.0001
EDSS at 10 years from onset (median, IQR)	1.5 (1.0–2.0; n = 20)	4.5 (3.0–6.5; n = 18)	0.0002
**EDSS annual deterioration (person-year)**			
Total	28/337 (8.3%)	20/229 (8.7%)	0.86
(With relapse)	11 (3.3%)	13 (5.7%)	0.16
(Without relapse)	17 (5.0%)	7 (3.1%)	0.29
**EDSS annual improvement (person-year)**			
Total	32/337 (9.5%)	4/229 (1.7%)	0.0001
(With relapses)	5 (1.5%)	2 (0.9%)	0.71
(Without relapses)	27 (8.0%)	2 (0.9%)	< 0.0001
**EDSS annually unchanged (person-year)**			
Total	277/337 (82.2%)	205/229 (89.5%)	0.0161
(With relapses)	22 (6.5%)	8 (3.5%)	0.13
(Without relapses)	255 (75.7%)	197 (86.0%)	0.0026

All EDSS scores were evaluated in the chronic phase more than 3 months after the last clinical episodes; the scores within 3 months from the last episodes were not used in this study.

*EDSS* Expanded Disability Status Scale, *IQR* interquartile range (25–75 percentile), *MS* multiple sclerosis, *NMOSD* neuromyelitis optica spectrum disorders, *SD* standard deviation.

### Progression of neurological disability in total

**Figure 1:**
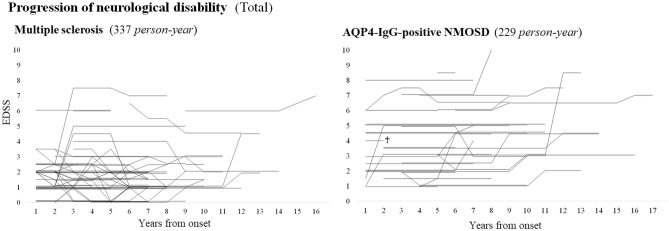
EDSS progression in each patient with MS or NMOSD. The cross-mark shows that the patient passed away because of malignancy. Patients with NMOSD are likely to show a stepwise progression of neurological impairment at each occasion of clinical attack, whereas patients with MS show gradual improvement or deterioration of neurological impairment irrespective of the relapse occurrence. *AQP4-IgG* anti-aquaporin-4 autoantibodies, *EDSS* expanded disability status scale, *MS* multiple sclerosis, *NMOSD* neuromyelitis optica spectrum disorders.

The progressions of EDSS in both disease groups by years from the onset, irrespective of the length of follow-up period or the occurrence of relapses, are shown in Fig. 1. In NMOSD group, most of the changes in EDSS took place as deterioration in neurological disability; in MS group, deterioration and improvement in EDSS score were similarly observed. As a consequence, during the whole follow-up period, the cross-sectional distribution of EDSS score was worse in NMOSD group than in MS group.

Within the initially enrolled patients, 53 MS patients were evaluated with EDSS at 5 years from the onset and 20 MS patients were evaluated at 10 years from the onset. In NMOSD group, all 31 patients were evaluated with EDSS at 5 years from the onset and 18 patients were evaluated at 10 years from the onset. The distributions of EDSS in MS and NMOSD groups at 5 and 10 years from the onset are listed in the middle of Table 1. The score of EDSS was much worse in NMOSD group than in MS group both at 5 years and 10 years from the onset.

### Impact of relapses to the progression of neurological disability

Data regarding to the relationship between attacks and the progression of irreversible neurological disability, irrespective of relapses, are summarized in the lower half of Table 1. Within the 20 occasions of EDSS annual deterioration in NMOSD patients, 13 occasions (65.0%) took place at the timing of relapses. Meanwhile, within the 28 occasions of EDSS deterioration in MS patients, 11 occasions (39.3%) took place at the timing of relapses.

When focusing on the period without clinical relapses, 255 (85.3%) of the 299 person-years of follow-up in MS group showed unchanged EDSS score, whereas 197 (95.6%) of the 206 follow-up years in NMOSD group showed unchanged EDSS score (p = 0.0002, Fisher’s exact test). In other words, neurological disability in NMOSD hardly changes without clinical attacks, whereas that in MS is more likely to change without relapses.

**Figure 2:**
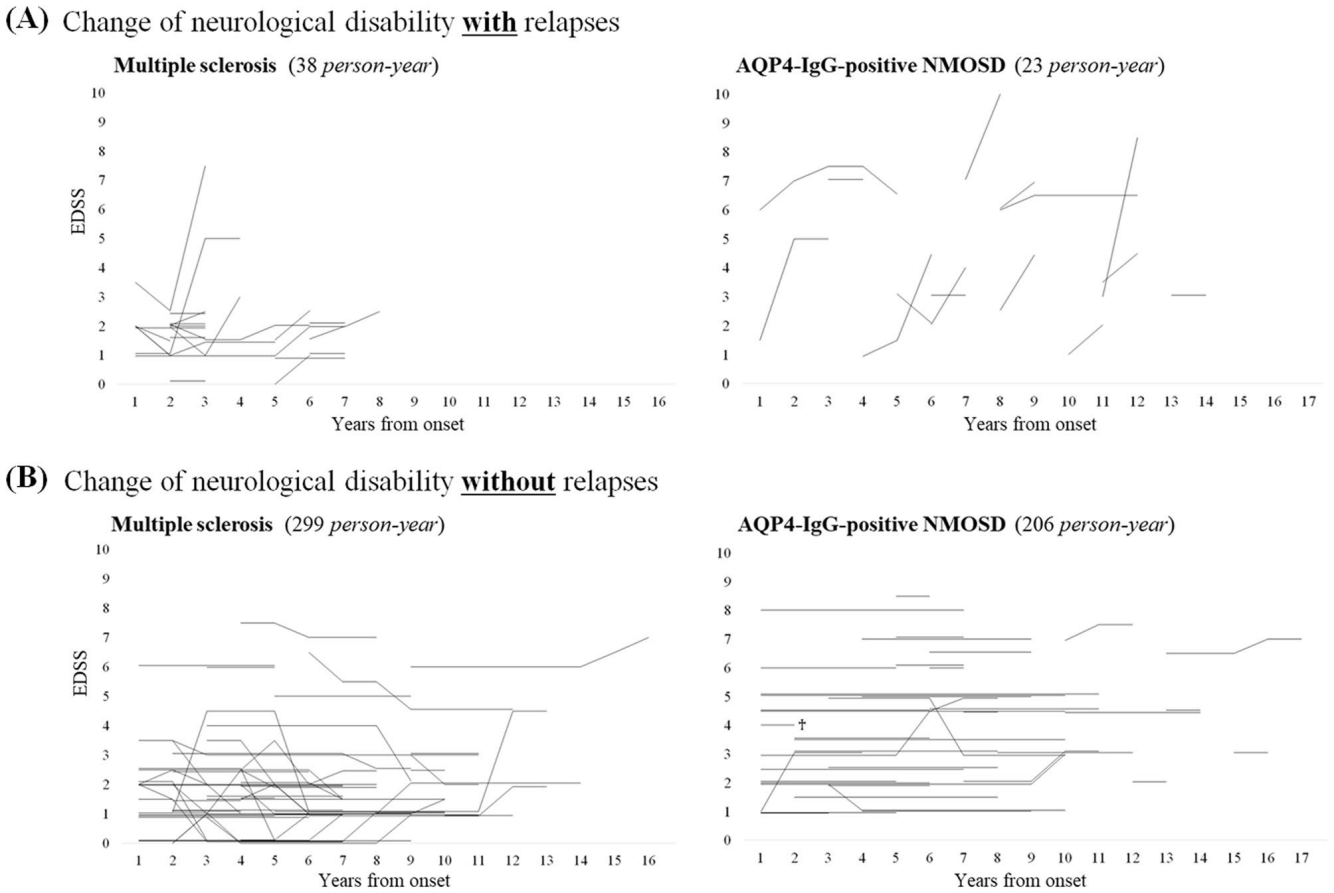
EDSS progression by relapse occurrence in MS and NMOSD. Neurological disability did not change without attacks in NMOSD, whereas it often spontaneously improved or deteriorated irrespective of the relapses in MS. Consequently, suppressing attack occurrence is the most important objective in NMOSD, whereas facilitating spontaneous improvement could be one of the possible therapeutic strategies in MS. *AQP4-IgG* anti-aquaporin-4 autoantibodies, *EDSS* expanded disability status scale, *MS* multiple sclerosis, *NMOSD* neuromyelitis optica spectrum disorders.

To visually confirm the difference in the impact of relapse to neurological disability between MS and NMOSD, we depicted line graphs of chronological change in EDSS for each patient by the relapse occurrence as shown in Fig. 2. As described above, relapses were more likely to be accompanied by EDSS deterioration in NMOSD than in MS (Fig. 2A). During the period without relapses, EDSS did not change at all in almost all NMOSD patients, whereas EDSS was more likely to change without relapses in MS patients (Fig. 2B).

### Spontaneous improvement of neurological disability in MS

As can be seen in the figures, not a few MS patients with proper treatments with DMDs showed a spontaneous EDSS improvement during their clinical course irrespective of relapse occurrence. The frequency of EDSS annual improvement in MS and NMOSD patients is shown in the bottom of Table 1. Improvement in EDSS was observed in only a few occasions in NMOSD patients. Meanwhile, EDSS annual improvement was observed in 32 occasions of the followed 337 person-years, which was much higher than that in NMOSD patients (p = 0.0001, Fisher’s exact test).

In MS group, the prevalence of minimally clinically important difference in EDSS score in the first 5 years was evaluated. Based on a previous literature, minimal clinically important difference was defined as 1.0 point change when the EDSS score was 0–5.0, and as 0.5 point change when the EDSS score was 5.5–8.5^20^. As a result, there were 12 patients whose EDSS improved with clinically important difference in the first 5 years from the onset, and 7 patient whose EDSS deteriorated with clinically important difference in the first 5 years from the onset. The characteristics of these two groups are summarized in Table 2. The baseline level of EDSS score was not significantly different between the improved group and the deteriorated group, but the number of cerebral lesions at 5 years from the onset (e.g. periventricular, subcortical) and the lesion site that is responsible for the neurological disability were significantly different between the groups. The number of cerebral lesions was significantly higher in the deteriorated group than in the improved group. The responsible lesion site was mainly located in the spinal cord in the improved group, whereas that was mainly located in the cerebrum (p = 0.0063) or brainstem (p = 0.0090) in the deteriorated group. The type of DMDs was not different between the two groups. For reference, the sites of myelitis that was responsible for the neurological disability in the improved group were cervical in 5 patients and thoracic in the other 5 patients.

**Table 2:** Patients with MS who showed clinically important difference in EDSS score in the first 5 years.

	Improved group	Deteriorated group	p
n (male:female)	12 (4:8)	7 (2:5)	–
Follow-up year	5.6 ± 2.1	5.9 ± 1.6	0.77
Onset age	28.8 ± 7.8	22.7 ± 6.0	0.0911
OB positivity	6/8 (75%)	6/6 (100%)	0.47
IgG-index	0.93 ± 0.30	0.80 ± 0.17	0.34
Findings at 5 years from the onset (*median, IQR)			
Relapses in the first 5 years*	0 (0–1)	1 (0–3)	0.26
Number of cerebral lesions*	2 (1–7)	14 (13–18)	0.0003
WML volume (cc)	3.8 ± 3.1	27.4 ± 8.7	0.0004
Grey matter volume (cc)	892 ± 62	899 ± 29	0.84
EDSS*	1.0 (1.0–2.0)	1.0 (1.0–4.0)	0.70
**Primarily responsible site of lesion for neurological disability (n)**			
Cerebral	2/12 (16.7%)	6/7 (85.7%)	0.0063
Optic nerves	0/12 (0.0%)	1/7 (14.3%)	0.37
Brainstem	0/12 (0.0%)	4/7 (57.1%)	0.0090
Myelitis	10/12 (83.3%)	2/7 (28.6%)	0.0449
**Relapse preventive therapies (n; allowing duplication)**			
IFN-beta	10/12 (83.3%)	7/7 (100.0%)	0.51
Fingolimod	8/12 (66.7%)	4/7 (57.1%)	1.00

Clinical data between the MS patients who showed clinically important improvement in EDSS over 5 years follow-up (i.e. improved group) and those who showed clinically important deterioration in EDSS over 5 years follow-up (i.e. deteriorated group) was compared. Minimal clinically important difference in EDSS score was defined as 1.0 point change when the EDSS score was < 5.5, and as 0.5 point change when it was 5.5–8.5. Brain volumetry was performed using a volumetric program offered by Icometrix (Leuven, Belgium).

*EDSS* expanded disability status scale, *IFN* interferon, *IgG* immunoglobulin-G, *IQR* interquartile range (25–75 percentile), *OB* oligoclonal band, *WML* white matter lesion.

*median (interquartile range [IQR]).

## Discussion

Based on the results of this study, progressive pattern of neurological disability in MS and NMOSD was suggested to be largely different. Progression of EDSS in NMOSD mainly took place at the timing of each attack occurrence, and it did not deteriorate for long time if there is no attack occurrence. Meanwhile, progression of EDSS in MS mainly took place irrespective of the relapse occurrence as previously known^21,22^. More importantly, EDSS in MS patients with DMDs often showed spontaneous sustainable improvement, which was hardly observed in the NMOSD patients. Such spontaneous EDSS improvement was likely to be seen in MS patients with neurological impairment based on myelitis and with less cerebral lesions.

One notable finding of this study was that neurological impairment in MS could be spontaneously improved, suggesting that damages in oligodendrocytes could be partially reversible or replaceable. Such spontaneous improvement was mainly seen in MS patients with responsible lesions in the spinal cord, whereas the MS patients with responsible lesions in the cerebrum or brainstem did not show such spontaneous remission. It is widely known that MS patients with more cerebral lesions and larger cerebral lesions are likely to show accelerated neurological disability and faster rate of global brain atrophy^23–25^. It may be better in upcoming clinical trials to distinguish the MS patients with only a few cerebral lesions, who are expected not to show EDSS deterioration in the following several years, and those with much more cerebral lesions, who are likely to show EDSS deterioration in the following several years.

The absence of spontaneous change in EDSS score independent from attacks in NMOSD patients supports the importance of taking the preceding relapse frequency in the last several years into consideration at the enrollment of clinical trials with NMOSD patients^26^. Also, this study indicated that one of the most appropriate primary outcomes to be evaluated in trials with NMOSD patients is the subsequent attack frequency or the severity of neurological damage in each attack, as neurological disability in NMOSD shows stepwise accumulation as a result of attacks^27^. Meanwhile, in clinical trials in MS, relapse frequency would not be among the most important enrollment criteria or outcomes to be evaluated, because EDSS improvement and deterioration often take place irrespective of the relapse occurrence. Suppression of neurological disability progression irrespective of relapses or facilitation of spontaneous EDSS improvement would be more desirable therapeutic outcome in MS patients. Since not a few MS patients showed spontaneous improvement in neurological disability, the achievement rate of EDSS recovery may be another possible outcome to be evaluated in the future clinical trials in MS.

There are several limitations in this study. First, almost all of the enrolled patients were Asian, except for one Caucasian female MS patient. Thus, whether the suggested spontaneous EDSS improvement in MS patients with DMDs can be also observed in MS patients of Caucasians and African-Americans is to be elucidated in the future clinical researches. Second, almost all of the enrolled patients were treated with standard relapse preventive therapies. Thus, the natural progressive patterns of neurological disability without any treatments are not known. Moreover, most of the studied MS patients in this study changed DMDs from IFN-β to fingolimod during the follow-up period. Thus, whether different types of DMDs may produce different rates of spontaneous remission in MS patients or not is unconcluded. However, such possibility seems to be unlikely between IFN-β and fingolimod, as there was no significant difference in the progression of neurological disability between these two drugs in a previous randomized double-blind study^28^. Lastly, we did not study EDSS progression in patients with serum anti-MOG antibody, which is expected to be elucidated in the future researches.

## Conclusions

NMOSD patients with relapse-preventive therapies show EDSS progression exclusively at the timing of each clinical attack, and EDSS does not deteriorate without attacks, suggesting that preventing clinical attacks is the most important therapeutic target in NMOSD. Meanwhile, MS patients treated with DMDs often show spontaneous improvement or deterioration in EDSS independent from relapses. Because the neurological disability in MS is reversible to some extent, therapeutic strategies to facilitate such spontaneous improvement in MS would be a promising therapeutic strategy in the future.
